# Annotations

**Published:** 1896-06-27

**Authors:** 


### Annotations.

### House Drains and Disease at Bow.

A house well drained is like a fort well garrisoned; it keeps danger and death at arm's length. In his annual report for 1894 Dr. Russell Main Talbot, medical officer of health for Bow, wrote: "It may be mere coincidence that the death rate has shown a large decrease directly after the appointment of an additional sanitary inspector." But that it was no mere coincidence is shown by the report for 1895, just published. For although the general death-rate at Bow was somewhat higher in 1895 than in 1894, the rate from zymotic diseases—that is, diseases due, among other things, to defective drainage—continued to maintain the same low rate as was experienced after the appointment of an additional sanitary inspector in 1894. This is an object lesson of a homely kind which Dr. Main Talbot does well to emphasise, for his employers in so thickly populated and comparatively poor a district as Bow, and other medical officers of health in similar districts, may with advantage mark the great practical utility of even one additional sanitary inspector in a very large and thickly populated area. Dr. Talbot tells us that the death-rate of Bow is not higher than that for the whole metropolis, and in 1895 was only 19·7 per 1,000. This is really a striking fact when it is remembered what the rate in such districts used to be before the era of modern sanitation opened. Civilization has its drawbacks, assuredly; but it most certainly has also its triumphs; and among these are the noble care that is now taken of the poor when they are sick, and the means employed to prevent them from falling into the ranks of sickness. That Bow should show a much lower death-rate than Manchester, or Hull, or Glasgow, is indeed a sanitary triumph for the despised "East End."

### Cycling and Heart Disease.

A series of articles on some of the medical aspects of cycling have appeared in a contemporary from the pen of Mr. E. B. Turner, F.R.C.S. They are of great importance, and will well repay study. It is calculated that more than a fourth of our adult population "cycles" or meditates cycling. Of this fourth a very considerable proportion have reached or passed middle age. It cannot but be that a number of these are the victims of "heart disease." What is the effect of cycling upon a person with a heart affection? The answer is that everything depends upon the nature of the affection. We have long ceased to regard all heart affections as of an identical degree of seriousness, and long left off the unscientific practice of wrapping all victims of heart disease in metaphorical cotton wool. It is now understood that most sufferers from cardiac trouble profit by exercise, and that some are advantaged by a good deal of exercise, and that of a vigorous kind. Cycling, according to Mr. Turner, whilst dangerous in affections of the aortic valves, is often of great service in uncomplicated mitral disease. Of course it must be cycling in moderation. Hill climbing and fast riding are peremptorily excluded, as is also riding which causes an approach to breathlessness. The great point for the beginner in such cases is, we hold, that he should spend adequate time and money in preliminary tuition, and not be in too great a hurry to be "off on his own account." Whilst on this subject we cannot but express surprise at the general incompetence and want of intelligence of the average "cycle" tutor. As a rule he is one of the stupidest creatures breathing. There would appear to be an excellent opening for both men and women tutors in this new amusement and recreation. Cycling has evidently "come to stay," and if a certain number of moderately-educated men and women with a little knowledge of physiology would take up the subject of tuition they would probably earn fair incomes, and certainly confer advantages on a good many people in the shape of increased comfort and improved health.

### Rate-supported Hospitals.

A letter which Dr. Sibley has written to the *Saturday Review* shows how easy it is to pick holes in the voluntary system on which the hospitals of this country are managed and supported. Of course there are certain defects in our hospital system, but what is there in heaven or on earth that exposes no weak spot to the sarcasm of the critical? It should, however, be remembered that exposure of deficiencies, while good argument for reform, is no argument in favour of revolution. There can be no doubt that State control of hospitals would, in the end, mean that the State would have to support them. It is impossible to believe that voluntary contributions would be forthcoming for the support of what would be really a Government department, any more than voluntary contributions now flow into the coffers of the Chancellor of the Exchequer; and those who are crying aloud for Parliamentary power to govern and control the great charitable hospitals of this country ought to face the fact, and proclaim it as part of their creed, that the result must be to throw the hospitals on the rates. Are we willing, then, to face this result and all that it entails? Before we accept the abolition of voluntaryism we ought to inquire whether the State-supported hospitals which we already possess are any more free from abuse, or any less open to criticism in regard to their administration, than the voluntary hospitals on which so much cheap sarcasm is expended; and we certainly ought to pay some regard to the income we should be throwing away by discouraging the voluntary system. We think we have heard of abuses even in rate-supported workhouse infirmaries; and, as to the money question, we find that, according to "Burdett's Hospitals and Charities," the income of the hospitals in Great Britain and Ireland amounted in 1894 to £2,184,076, all of which was from voluntary contributions, and may be taken as in some sense a measure of the burden which it is proposed to throw upon the ratepaper. It seems to us that to refuse such a valuable source of income would be an unjustifiable waste, while Government control has so far shown itself a measure of doubtful efficacy.

